# Impact of Lipid and ASCVD-Modulating Agents on Glycemia and New-Onset Diabetes or Prediabetes

**DOI:** 10.1016/j.jacadv.2025.101700

**Published:** 2025-04-16

**Authors:** Om P. Ganda

**Affiliations:** a Clinical Research and Adult Diabetes Sections, Joslin Diabetes Center, Boston, Massachusetts, USA; b Department of Medicine, Beth-Israel Deaconess Medical Center, and Harvard Medical School, Boston, Massachusetts, USA

**Keywords:** antiinflammatory drugs and diabetes, ASCVD and diabetes, lipid drug associated diabetes, lipid drugs

The effects of lipid-lowering drugs on glucose metabolism and the emergence of new-onset diabetes (NOD) have been a matter of concern. Since the prestatin era, nicotinic acid has been known to induce or exacerbate hyperglycemia. This was confirmed in recent randomized controlled trials (RCTs), notably AIM-HIGH (Atherosclerosis Intervention in Metabolic Syndrome With Low HDL/High Triglycerides) and HPS-THRIVE (HEART Protection Study 2-Treatment of HDL to Reduce the Incidence of Vascular Events). This and other adverse events,^1^ and the overall lack of cardiovascular event (CVE) benefits, have led to its very limited use.

Currently, statins remain the drug of choice for CVE reduction. A recent comprehensive review by CTT (Cholesterol Treatment Trialists’) consortium provides the incidence of NOD or worsening glycemia from 23 placebo-controlled RCTs (n = 156,000, median follow-up >4.0 years), of which ∼20% had pre-existing diabetes mellitus (DM).^2^ The rate ratio (RR) for NOD with low/moderate intensity statin and high intensive statin were 10.0% (95% CI: 1.04-1.16) and 36% (95% CI: 1.25-1.48), respectively. Of those who developed NOD, 62% were in the top quartile of glycemia at baseline. However, the mean increase in A1C was 0.06 and 0.08%, respectively. In those with pre-existing DM, the RR for worsening glycemia with low/moderate and intensive therapy groups were 10% (95% CI: 1.06-1.40) and 24% (95% CI: 1.06-1.44), respectively. The precise mechanism of hyperglycemic effects of statins is not firmly established, and likely involves both insulin secretion and its peripheral actions. While the emergence of NOD with statins was still associated with favorable reductions in CVEs, many patients and care providers remain concerned about the statin-induced NOD, particularly in primary prevention. This is also a relevant issue when choosing add-on lipid therapy when maximally tolerated statins do not provide optimal low density lipoprotein-cholesterol (LDL-C), apo-B, and non-high density lipoprotein-cholesterol (HDL-C) goals. Therefore, choosing any add-on therapy with neutral or favorable effects on hyperglycemia, while promoting additional reductions in CVEs and no major adverse effects, are worthy of consideration. Furthermore, as the prevalence of obesity and DM continue unabated and still on the rise, this becomes a particularly relevant issue for millions of people globally.

Several lipid-lowering agents are associated with generally neutral effects on glycemia, while improving CVEs in combination with statins. In the IMPROVE-IT (Ezetimibe added to Statin Therapy after Acute Coronary Syndromes) trial, simvastatin 40 mg plus ezetimibe, compared to simvastatin alone, was associated with a neutral impact on glycemia, or modestly lowered risk of NOD according to some recent reports.^3^ Of particular interest, the statin-ezetimibe combination was associated with more striking reduction in CVEs in people with DM (n = 5,284 of 18,144), compared to nondiabetics.^4^ Ezetimibe is currently the most common add-on drug to statin when LDL-C goal is not achievable with statin alone. More recently, neutral effects on glycemia were observed with bempedoic acid, an ATP-citrate lyase inhibitor, acting upstream to the statin site of inhibition in the cholesterol synthetic pathway. In that large RCT (Clear Outcomes) in people with a history of statin intolerance, there was 21.1% reduction in LDL-C, leading to 13% reduction in CVE, and no significant effects on glucose or A1C in those with preexisting prediabetes or normoglycemia.^5^^,^^6^ Interestingly, in the primary prevention subgroup in that trial (n = 4,206) had even greater reduction in CVE (30%), compared to the secondary prevention group. Of another major LDL-lowering class, PCSK9 inhibitors (evolocumab and alirocumab) are also devoid of adverse effects on glycemia, while providing the best efficacy for LDL-C reduction in combination with statins in both RCTs.^7^^,^^8^

In the prestatin era, bile acid sequestrants were also a drug of choice for LDL-C reduction. Both cholestyramine and colesevelam are associated with a modest A1C reduction of ∼0.5% along with 13% to 17% decrease in LDL-C, while modestly increasing triglycerides (TG).^9^ Their favorable glycemic effects may be partly mediated via increased luminal bile acid concentration leading to activation of incretin-mediated effect via a G-protein-coupled receptor. Currently, colesevelam remains Food and Drug Administration-approved for both hyperglycemia and LDL-C management, but currently superseded by many better-tolerated options for either.

Looking into glycemic effects of currently used TG-lowering drugs, a variety of studies have reported variable results with fibrates. Among the class, those with predominantly peroxisome proliferator-activated receptor (PPAR)-¥ activity, such as bezafibrate and fenofibrate, generally tend to moderately lower glucose and A1C. In contrast, pemafibrate has predominantly PPAR-α activity, which might reflect its lack of glucose-lowering effect, while the TG-lowering effect is similar or greater with PPAR-α activity.^10^ In PROMINENT (Pemafibrate to Reduce Cardiovascular Outcomes by Reducing Triglycerides in Patients with Diabetes) trial with pemafibrate, the largest fibrate trial in people with pre-existing DM, no glycemic benefits were reported despite TG reduction. Consistent with this, among thiazolidinediones, pioglitazone, a dual PPAR-α/-¥ agent, had more potent TG-lowering effect, as compared to rosiglitazone, a predominantly PPAR-¥ agent in a head to head trial of these thiazolidinediones.^11^ Pioglitazone, as a glucose and TG-lowering agent, has been underutilized due to concerns with weight gain and risk of HF.

The only approved drug to add to statin in people with high TG and high risk of atherosclerotic cardiovascular disease to reduce CVEs is the omega-3 fatty acid (OM3FA), specifically IPE (icosapent ethyl). In the largest RCT in such a population, the REDUCE-IT (Reduction of Cardiovasculr Events with Icosapent-Ethyl-Intervention Trial), there were neutral effects on glycemia in the nondiabetic subjects with metabolic syndrome (35% of the total cohort).^12^ Some studies in subjects with impaired glucose regulation reported significant improvement in glycemia, or insulin resistance, with mixed OM3FAs.^13^ Some of the variable results in various studies on this issue may possibly relate to differences in OM3FAs composition, dosing, and proposed effects on microbiome.

With a renewed interest in the lipid effects and CVE reduction with the novel cholesterol ester transfer protein (CETP) inhibitor obecetrapib, undergoing a large RCT, its potential effects on NOD and glycemia are eagerly awaited. Obecetrapib has the largest effects on lowering LDL-C, and apo B, while it is most potent in raising HDL-C, compared to all others in this class. Among the older agents, anacetrapib (REVEAL [Randomized Evaluation of the Effects of Anacetrapib through Lipid Modification]) was the largest, and it reported significant reduction in new DM. In a meta-analysis of 4 RCTs with CETP inhibitors including REVEAL, there was a significant reduction in new DM (OR: 0.88; 95% CI: 0.81-0.96; *P* = 0.005).^14^ The mechanism, and any relationship with the largest increase in HDL-C with obecetrapib in this class, will be of much interest.

Among the other novel agents undergoing trials with RNA-based therapies for high TG and mixed dyslipidemia, angiopoietin-like protein (ANGPTL)-3 and 4, inhibitors of LPL, are associated with favorable glycemic effects in short-term efficacy and safety trials.^15^ Examples include vupanorsen, zodasiran, evinacumab, and others. Also awaited are glycemic effects with apo C-3 inhibitors, also acting via LPL inhibition, currently in RCTs for very high TG as well as mixed dyslipidemia. Such novel agents to improve TG management, in addition to fibrates and OM3FAs, are much needed.^16^ Examples include volanesorsen, olezarsen, plozasiran, and others.

A class of anti-inflammatory agents, interleukin-1ß inhibitors, were also studied for their glycemic effects. They reduce CVEs, despite no effects on lipids. In the CANTOS (Canakinumab Antiinflammatory Thrombosis Outcomes Study) trial, canakinumab had neutral effects on NOD, while CVEs were reduced. However, almost 90% of 10,016 subjects had DM or pre-DM at baseline. In an earlier small trial with another agent in the class, anakinra, there were favorable effects on beta-cell function, insulin sensitivity, and A1C levels.^17^ None of these agents are approved for CVE reduction due to associated adverse events, while they are approved for some atypical forms of arthropathy.

The Table 1 provides a brief overview of current or emerging lipid-lowering or atherosclerotic cardiovascular disease-modulating agents on potential glycemic and diabetogenic effects.

**Table 1:** Potential Effects of Lipid and ASCVD-Modulating Agents on Glycemia and New-Onset Diabetes or Prediabetes

Increase	Neutral	Decrease
HMG-CoA reductase inhibitors (statins)	Ezetimibe	Bile-acid sequestrants (cholestyramine, colesevelam)
Nicotinic acid	Bempedoic acid	PPAR-¥ and dual PPAR-α/-¥ agents (fibrates, TZDs (pioglitazone, rosiglitazone)
	PCSK-9 inhibitors (evolocumab and alirocumab)	CETP inhibitors (evacetrapib, anacetrapib, obecetrapib)
	PPAR-α agonists, eg, pemafibrate	ANGPTL-4 inhibitors
	Omega-3 fatty acids (icosapent-ethyl)	ANGPTL-3 inhibitors (limited data)
	IL-1ß inhibitor (canakinumab)	IL-1ß inhibitor (anakinra)
	Apo C-3 inhibitors (limited data)	

ANGPTL = angiopoietin-like protein; ASCVD = atherosclerotic cardiovascular disease; HMG-CoA = hydroxymethylglutaryl-coenzyme A-reductase Inhibitor; IL = interleukin; TZD = thiazolidinedione.

## Conclusions

Statins remain by far the preferred agent in the prevention of CVEs. However, since the initial reports of increased incidence of NOD with statins, albeit modest, there is some concern regarding their use in primary prevention of CVEs, particularly with intensive dosing. Moreover, in many people, current lipid goals are not achievable with statins. Currently, several nonstatin options with neutral and favorable glycemic effects exist. The Mendelian randomization studies suggested that based on their genetic targets, ezetimibe, and PCSK-9 inhibitors, like statins, might be diabetogenic. However, this has not been borne out by clinical trials with these agents. Interestingly the ATP-citrate lyase, the target for bempedoic acid, predicts a neutral effect on glycemia, now confirmed in clinical trials. From the practical standpoint, it is reassuring that each of these 3 classes of drugs is proving to be neutral with regard to their glycemic effects. Among the CETP inhibitors, the emerging data with obecetrapib, the most potent in this class, are encouraging, while the cardiovascular event trials are underway. Finally, on-going trials with apo C-3 and ANGPTL inhibitors in people with hypertriglyceridemia are also awaited.

## Funding support and author disclosures

Dr Ganda has received a periodic fee from Arrowhead Pharma as a member of the Data Monitoring Board; and also received consulting honorarium from Amsterdam Pharma.
